# Right–left digit ratios, a novel form of asymmetry: Patterns of instability in children and relationships to platelet counts and hospitalization in adults with COVID-19

**DOI:** 10.3389/fpubh.2022.995025

**Published:** 2022-10-14

**Authors:** Anna Kasielska-Trojan, John T. Manning, Maciej Jabłkowski, Jolanta Białkowska-Warzecha, Oliwia Kwasniewska, Angelica L. Hirschberg, Bogusław Antoszewski

**Affiliations:** ^1^Plastic, Reconstructive and Aesthetic Surgery Clinic, Institute of Surgery, Medical University of Lodz, Lodz, Poland; ^2^Applied Sports, Technology, Exercise, and Medicine (A-STEM), Swansea University, Swansea, United Kingdom; ^3^Department of Infectious and Liver Diseases, Medical University of Lodz, Lodz, Poland; ^4^Medical University in Lodz, Lodz, Poland; ^5^Department of Gynecology and Reproductive Medicine, Karolinska University Hospital, Stockholm, Sweden; ^6^Department of Women's and Children's Health, Karolinska Institute, Stockholm, Sweden

**Keywords:** digit ratio (2D:4D), fluctuating asymmetry, digit 5, COVID-19, hospitalization

## Abstract

High right minus left (R-L) asymmetry of digit ratios has been reported to be linked to hospitalization for COVID-19. Here we examined the developmental patterns of this novel form of asymmetry in children and further explored their relationships to platelet counts and hospitalization for COVID-19 in adult patients. We considered ratios calculated from four digits (2D, 3D, 4D, 5D) in: (i) a sample of healthy participants aged 2 years to 18 years (*n* = 680, 340 males) and (ii) 96 adult patients (42 males) hospitalized for COVID-19 and 100 controls (53 males). The protocol for (ii) included a questionnaire and laboratory test results. In sample (i) of the six unsigned digit ratio asymmetries, those which included 5D had the highest mean asymmetry with the greatest between-individual variation and they were unstable over the age range of 2 years to 18 years. In sample (ii) patients showed higher asymmetries than controls in four ratios (2D:4D, 2D:5D, 3D:5D, 4D:5D) and a sum of asymmetries of the two independent ratios (2D:4D+3D:5D) correlated positively with platelet counts and hospitalization. Conclusion: Means and SDs of digit ratio asymmetry that include the 5th digit are high and age-unstable. Digit ratio asymmetry, particularly 5th digit ratio asymmetry and a composite measure of 2D:4D + 3D:5D asymmetry, may be positively linked to high platelet counts in COVID-19 patients and to an elevated risk of hospitalization.

## Introduction

COVID-19 is a mild disease in most patients but in some it progresses to acute illness and hospitalization. Therefore, in order to inform effective public health measures, it is of importance to be able to identify patients who are at elevated risk of progression to severe illness. Kasielska-Trojan et al. (1) have suggested that high digit ratio asymmetries (putative markers of postnatal stress and consequent developmental instability) are linked to a tendency to develop more severe COVID-19. They considered digits two to five (2D, 3D, 4D, 5D), calculated six ratios (2D:3D, 2D:4D, 2D:5D, 3D:4D, 3D:5D, 4D:5D) and focused on links between COVID-19 and the unsigned Right-Left asymmetries of each ratio (|R-L|). Hospitalized patient and control means for unsigned asymmetries were compared in 54 patients and 100 controls. Patients had greater unsigned asymmetry than controls for all ratios that included 5D (i.e. 2D:5D, 3D:5D, 4D:5D) and also for 2D:4D. The two ratios with the largest effect sizes for these patient–control differences were 2D:4D and 3D:5D and together their composite asymmetry gave a patient–control effect size of *d* = 1.04 (1).

Unsigned digit ratio asymmetry is a novel trait. In contrast, asymmetry of paired body traits (FA) has been the subject of much research related to health and fertility. In humans, elevated levels of FA are associated with reduced ejaculate size and poor quality of sperm (2), smaller family size (3, 4), high resting metabolic rate (4, 5), elevated weight and BMI (6, 7), schizophrenia (8), attention deficit disorder (9), developmental delays in childhood (10), Down's syndrome (11) and breast cancer (12–15). For reviews see Thornhill and Moller (16) and Benderlioglu (17). Relative asymmetry of single digit lengths and mean digit ratios in humans (2D:3D, 2D:4D, 2D:5D, 3D:4D, 3D:5D, 4D:5D) have been considered across age groups 2 years to 18 years (18, 19) and in adult mice (20). For humans the magnitude of asymmetry was greatest in very young children. It then reduced with age in a pattern that was closely related to a reduction in growth rate and metabolic rate. This pattern varied in strength across the digits with the strongest reduction in absolute and relative asymmetry found for 5D. Digit ratios also showed reductions across age groups with the greatest magnitude of reductions seen in ratios which included 5D.

Body asymmetry has been reported to be correlated with a number of diseases. |R-L| breast size and dermatoglyphic ridges have been linked to breast cancer (13) for the former and schizophrenia (16) for the latter. With regard to |R-L| differences in digit length, these asymmetries are negatively correlated with markers of fertility such as sperm counts and sperm viability (2). However, in this regard, unsigned asymmetries consist of |R-L| differences in single paired traits such as length of the 2nd digit (|right 2nd digit–left 2nd digit|) corrected for mean trait size (referred to as relative unsigned asymmetry). Correlations between relative asymmetry of single traits such as 2nd digit length and ejaculate size are weak. However, summing across asymmetries from more than one digit increases effect sizes. In contrast to single trait relative asymmetry, unsigned |R-L| digit ratio asymmetry includes information from two rather than one trait. For example, for 3D:5D we have: (|right 3D:5D–left 3D:5D|). In common with the more conventional single trait of relative asymmetry, unsigned digit ratio asymmetry may be summed across digit ratios. These summed asymmetries may increase predictive power when considering associations with hospitalization or with the results of laboratory tests.

The study of Kasielska-Trojan et al. (1) was limited by lack of knowledge concerning the properties of unsigned digit ratio asymmetries and by the relatively small sample of patients (1). The latter meant that associations between patient asymmetries and laboratory tests might be unreliable. Properties and the possible practical role of digit ratio asymmetries as markers of developmental instability have not been previously described in the literature. Such novel approach could provide simple clinical tool to determine subjects susceptible for diseases related to developmental instability. To provide such theoretical basis for our clinical study, first we decided to examine the mentioned aspects in a separate study and include it here as a background for the clinical part.

Therefore, the purpose of this present report was:

(i) Firstly, to consider patterns of unsigned digit ratio asymmetries (FAs) across a large sample of healthy participants aged from 2 to 18 years. Our focus in this was to examine differences in digit ratio asymmetries with particular emphasis on those that had been linked to COVID-19 hospitalization, i.e., ratios that included 5D (2D:5D, 3D:5D, 4D:5D) and the composite asymmetry that was composed of 2D:4D plus 3D:5D (Study I),
(ii) Secondly, to increase the sample size of the patients and focus on the relationships of their unsigned digit ratio asymmetries (particularly, those that included 5D and the composite asymmetry of 2D:4D plus 3D:5D) to laboratory test results (including platelet count) in addition to a measure of COVID-19 severity, i.e., hospitalization (Study II).

## Materials and methods

### Study population

To meet the aims, in study I and II different groups of participants from different centers (countries) were included (multicenter study).

#### Study I

The protocol for this study is given in detail by Wilson and Manning (18) and Manning (19). Briefly, digit lengths were recorded directly using steel calipers measuring to 0.01 mm. Measurements were made on the palmar surface of the hand, from the tip of the digit to the mid-point of the crease which was proximal to the palm. This crease approximates to a mid-point position on the proximal phalanx. Digit lengths for all five fingers were included but it proved difficult to determine the mid-point of the proximal crease for 1D. Therefore, we focused on digits 2D to 5D. Participants with one or more injuries to their digits were excluded from the sample. Measurements were repeated on 20 participants. Repeatability of the digit lengths were high, with intra-class correlation coefficients varying from r1 = 0.98, to r1 = 0.99, all *p* < 0.0001.

There were 680 Caucasian participants (340 males) aged from 2 to 18 years, 40 (20 males) participants per year group. All the children were resident in the North West area of England. For children younger than 18 years informed permission to measure was obtained from parents and play schools/schools etc. For the 18-year group informed permission was obtained from participants. Digit length measurements were made in inner-city and suburban pre-school groups, play centers, primary schools, secondary schools and sixth-form colleges (participants were chosen randomly, probability sampling). Permission for the Study was obtained from the Ethics Committee of the Faculty of Biological Sciences, University of Liverpool.

#### Study II

Patients with diagnosed COVID-19 who were hospitalized in the Department of Infectious Diseases and Liver Diseases of a Medical University due to the severe or high risk of severe COVID-19 during the first waves of COVID-19 (before vaccinations) were included [*n* = 96, complete measurements were available for *n* = 91: 42 men (mean age 59.8 ± 15.5 years) and 49 women (mean age 60.2 ± 15.4 years)]. These were consecutive patients who met study criteria and gave a conscious consent to participate, in the study period. The protocol of the study included a clinical questionnaire and anthropometric measurements. Eight measurements were taken from patients' hand photographs: digit lengths (2D, 3D, 4D and 5D) [right (R) and left hand (L)] and all six ratios were calculated to obtain unsigned asymmetries [|(right – left)|- Δ2D:3D, Δ2D:4D, Δ2D:5D, Δ3D:4D, Δ3D:5D, Δ4D:5D]. Further, we calculated composite asymmetry (cA) of all six ratios and a “Clinical Composite Asymmetry” (CCA) for the independent ratios of 2D:4D and 3D:5D. All measurements were made twice by AKT using the GNU Image Manipulation Program (GIMP) version 2.10.20 according to the standard protocol of measurements (7). Clinical data and laboratory tests (white blood count, procalcitonin, fibrinogen, d-dimers, platelet count) were obtained from medical charts. Controls (*n* = 100) included 47 women (mean age 51.3 ± 16.1 years) and 53 men (mean age 52.2± 14.4 years) with a negative history of COVID-19. The protocol was agreed by the Bioethical Committee of the Medical University of Lodz (RNN/152/20/KE).

### Statistical analysis

In Study I Shapiro-Wilk test was used to check the normality of distribution of the data. *T*-test and the Mann-Whitney test were used to assess whether the mean values of signed asymmetries differ significantly from zero. Spearman correlations were applied for six unsigned asymmetries relationships with age and to test relationships between individual unsigned digit ratios asymmetries.

In study II, analysis was conducted on the differences in digit ratios unsigned asymmetries between patients hospitalized due to COVID-19 and controls. The normality of distribution of the tested variables was examined (using Kolmogorov-Smirnov test) and the homogeneity of variances was checked (using the Bartlett test). Mann Whitney test was used to compare digit ratio FAs between groups. Analysis of variance (ANOVA) was used to check if observed correlations were independent of sex, also non-parametric tests were applied if ANOVA assumptions were not met. Effect sizes for inter-group differences were evaluated with Cohen's *d* for *t*-tests and omega-squared (ω2) for ANOVA. The interpretation of descriptors of magnitude for *d* were: small >0.20, medium >0.50 and large >0.80 and for ω2 > 0.01 —weak, > 0.06—medium, > 0.14—strong effects. Additionally, differences in composite FAs were analyzed (sum of all digit ratios FAs -cFA) and Clinical Composite Asymmetry (CCA) with Mann-Whitney test and ANOVA controlled for sex (logistic regression). The quality of predictor (CCA) was characterized by the area under the ROC curve (AUC). Additionally, the optimum cut-off point (OCP) was determined, for which sensitivity and specificity as measures of prediction quality were the highest. Spearman rank analyses were used to evaluate correlations between the CCA and clinical parameters (severity of COVID-19, days of hospitalization, days of oxygen therapy, laboratory test on admission–platelet count, white blood count, d-dimers, fibrinogen). In order to remove the effects of sex, age and BMI on the associations, FAs were then log-transformed to normal distribution and parametric tests (multiple logistic regression analyses–standardized regression coefficients) were used for associations with platelet count (significant in non-parametric analysis). The probability of p < 0.05 was accepted as a level of significance.

## Results

### Study I

#### Signed digit ratio asymmetries

The signed digit ratio asymmetries had low values for skewness indicating their distributions were symmetrical as expected in a normal distribution (skewness varied from 0.027 to 0.681). Kurtosis values were high and positive indicating leptokurtosis. Values ranged from 1.55 to 4.38 indicating the asymmetries were concentrated at the means of the distributions and not in the tails. “Ideal” fluctuating asymmetry has a mean of zero. There were small but significant deviations from zero (i.e. directional asymmetry: Right digit ratio>Left digit ratio). Notably they were found in 2D:4D together with 2D:5D and 3D:5D, and 4D:5D was also close to significance (*p* = 0.09) (see Table 1).

**Table 1:** Signed digit ratio asymmetries (Right digit A/Right digit B)–(Left digit A/Left digit B).

**One-sample** ***t*****-test (mean set at zero)**					
**Signed asymmetry**	**mean**	**t**	***p***	**Skewness**	**Kurtosis**
R-L 2D:3D	0.002	1.104	0.270	−0.401	3.129
R-L 2D:4D	0.005	2.675	0.008	0.452	3.219
R-L 2D:5D	0.011	3.619	0.0003	0.027	1.555
R-L 3D:4D	0.003	1.420	0.156	0.681	4.388
R-L 3D:5D	0.009	2.716	0.007	0.324	1.644
R-L 4D:5D	0.005	1.697	0.090	0.143	2.644

#### Unsigned digit ratio asymmetries

Unsigned digit ratio asymmetries take the form of a truncated “half-normal” distribution. Of the six asymmetries, those that included 5D had higher means and SD's (means 0.054 to 0.063, SD's 0.051 to 0.055) than asymmetries not including 5D (means 0.035 to 0.040, SD's 0.033 to 0.038). With regard to age-dependent changes in unsigned asymmetries, we found all asymmetries were negatively correlated with age. However, the strength of the correlations was not uniform with the strongest reduction seen in asymmetries of ratios that included 5D (rs from −0.243 to −0.298) compared to ratios not including digit five (rs from −0.154 to −0.166) (Table 2).

**Table 2:** Means, SDs for six unsigned asymmetries together with Spearman correlations for relationships with age.

**Unsigned R-L**	**Mean**	**SD**	***r_*s*_***	***p***
2D:3D	0.035	0.033	−0.161	< 0.0001
2D:4D	0.040	0.035	−0.166	< 0.0001
2D:5D	0.062	0.055	−0.298	< 0.0001
3D:4D	0.040	0.038	−0.154	< 0.0001
3D:5D	0.063	0.057	−0.279	< 0.0001
4D:5D	0.054	0.051	−0.243	< 0.0001

With regard to intercorrelations between asymmetries, such correlations were substantial (Table 3). Focusing on our two “groups” of correlations, i.e., those that include 5D and those that do not, we note that 3D:5D and 2D:4D were not significantly correlated. This may be of importance because information regarding developmental instability may be independent in these two ratios. Thus, it is of interest that a composite asymmetry score of 2D:4D + 3D:5D has been reported to be strongly correlated with probability of hospitalization for COVID-19.

**Table 3:** Correlations between individual unsigned digit ratios asymmetry using the Spearman correlation.

**Unsigned R-L**	**2D:3D**	**2D:4D**	**2D:5D**	**3D:4D**	**3D:5D**	**4D:5D**
2D:3D	1					
2D:4D	0.279 (< 0.0001)	1				
2D:5D	0.176 (< 0.0001)	0.264 (< 0.0001)	1			
3D:4D	0.184 (< 0.0001)	0.203 (< 0.0001)	0.072 (0.059)	1		
3D:5D	0.120 (0.002)	0.018 (0.644)	0.376 (< 0.0001)	0.268 (< 0.0001)	1	
4D:5D	0.014 (0.713)	0.141 (0.0002)	0.344 (< 0.0001)	0.198 (< 0.0001)	0.412 (< 0.0001)	1

Red color means significant correlations.

### Study II

**Figure 1:**
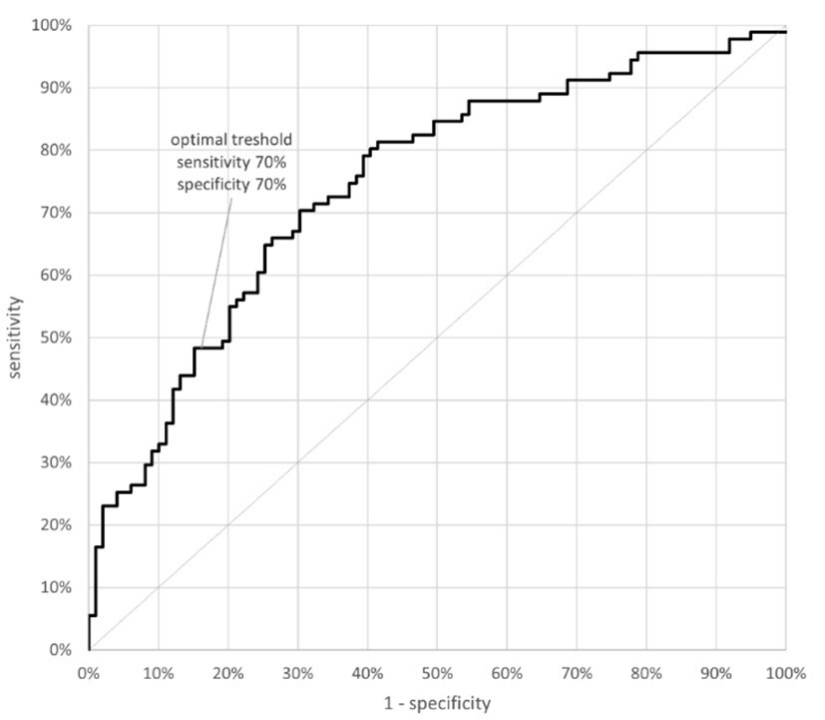
ROC curve for the clinical composite asymmetry predicting probability of hospitalization due to COVID-19.

Demographic and clinical characteristics of patients hospitalized for COVID-19 are shown in Table 4. Intra-observer reliability for all ratios for observer AKT was high: the coefficient of reliability for raw measurements (R) ranged from 96.07% (for 3D:4D L) to 99.66% (for 2D:5D R). Four unsigned digit ratio asymmetries differed between patients and controls with patients>controls (2D:4D, *d* = 0.5; 2D:5D, *d* = 0.6; 3D:5D, *d* = 0.7; 4D:5D, *d* = 0.5, Table 5). Two-factor ANOVA showed that the mean differences were independent of sex, with the following effect sizes (ω2): |Δ2D:4D| = 0.054; |Δ2D:5D| = 0.076; |Δ3D:5D| = 0.117; |Δ4D:5D| = 0.051. Composite asymmetry (cA - sum of all six ratios' asymmetry) was higher in the patients compared to controls (*d* = 0.77). Clinical Composite Asymmetry (CCA)- sum of the two “independent” asymmetries (2D:4D and 3D:5D) showed the highest effect size with patients>controls, *d* = 0.92 for women and *d* = 0.81 for men and the differences were independent of sex. The area under an ROC curve (AUC) was 0.744 (AUC = 0.5) with the cut-off point of 0.079. CCA higher than 0.079 discriminates hospitalized patients with a sensitivity and specificity of 70% (Figure 1). The risk of hospitalization in case of the index >0.079 is 2.5 times higher than in those with lower CCA (OR = 2.421).

**Table 4:** Demographic and clinical characteristics of patients hospitalized for COVID-19.

	**Patients**		**Controls**	
	**Women *n* = 49**	**Men *n* = 42**	**Women *n* = 47**	**Men *n* = 53**
Age [yrs, mean, SD]	60.2 ± 15.4	59.8 15.5	51.3 ± 16.1	52.2 ± 14.4
BMI [kg/m^2^, mean]	28.7	29.8	27.9	29.1
Smokers/Ex-smokers [*n* (%)]	4(8.2)/17(34.7)	12(28.6)/21(50)	2(4.3)/13(27.7)	12(22.6)/23(43.4)
• COVID-19 symptoms [*n*] • *Dyspnoea* • *Cough* • *Fever* • *Fatigue* • *Diarrhea* • *Other (loss of smell and taste, dyssomnia, headache, skin lesions)*	• • 30 • 40 • 43 • 33 • 11 • 5	• • 27 • 33 • 35 • 25 • - • 5	-	-
COVID-19 severity score* [mean] [Likert scale 1(mild) – 4 (critical)]	2.2	2.3	-	-
Length of hospitalization/oxygen therapy [days]	15.2/9.9	14.9/13.1	-	-

**Table 5:** Digit ratios' asymmetries in patients and the controls.

	**Patients (*n* = 91)**	**Controls (*n* = 100)**	**Mann-Whitney test**		
**Unsigned right-left ratio (FA)**	**mean±sd**	**mean±sd**	**Z**	***p***	***d* Cohen**
Δ 2D3D^a^	0.031 ± 0.02	0.027 ± 0.02	1.289	0.1975	0.156
Δ 2D4D	0.044 ± 0.03	0.029 ± 0.03	3.324	0.0009*	0.496
Δ 2D5D	0.086 ± 0.08	0.049 ± 0.04	3.668	0.0002*	0.594
Δ 3D4D^a^	0.032 ± 0.03	0.031 ± 0.03	0.018	0.9853	0.028
Δ 3D5D	0.080 ± 0.07	0.039 ± 0.03	5.311	< 0.0001*	0.734
Δ 4D5D	0.062 ± 0.06	0.038 ± 0.04	3.112	0.0019*	0.476

^a^99 controls, *significant differences.

CCA correlated positively with platelet count. It did not correlate with d-dimers, fibrinogen, procalcitonin and days of hospitalization or oxygen therapy as well as severity of the disease (Table 6). Additionally after removing the effects of sex, age and BMI, platelet count was significantly related in multiple regression tests (standardized regression coefficients) with asymmetries involving 5D: i.e., 2D:5D (*p* < 0.05) and 4D:5D (*p* < 0.05) but not with other ratios' asymmetries (2D:3D, 2D:4D, 3D:4D, 3D:5D, all *p* > 0.05).

**Table 6:** Spearman correlation coefficients for 2D:4D plus 3D:5D FAs and clinical parameters in patients with COVID-19.

**FA 2D:4D+ FA 3D:5D (CCA) vs**.	**N**	**R Spearman**	**t**	***p***
Severity^a^	86	−0.106	−0.976	0.3319
Hospitaliozation (days)	87	0.134	1.248	0.2154
Oxygen therapy (days)	70	−0.122	−1.011	0.3158
d-dimers	80	−0.002	−0.019	0.9853
Fibrinogen	42	−0.285	−1.883	0.0670
Platelet count*	78	0.254	2.285	0.0251
Procalcitonin	50	0.105	0.730	0.4687
White blood count	79	0.166	1.475	0.1443

^a^0–no symptoms; 1–mild; 2–medium, 3–severe, 4–critical, 5–death, *significant differences.

## Discussion

The presented studies aimed to determine patterns of digit ratio FAs across healthy participants (Study I) and to further investigate their relationships of hospitalization because of COVID-19 and patients' laboratory test (Study II). With regard to Study I, we have found that signed digit ratio asymmetry in healthy children showed a distribution that is close to normal with low skewness. There was significant positive kurtosis but this is also commonly found in the more conventional forms of “ideal” fluctuating asymmetry (21). However, there was also evidence of directional asymmetry in |R-L| asymmetries of 2D:4D and in asymmetries of ratios that include 5D. Moreover, with regard to unsigned asymmetries of the latter, they showed elevated means and SD's and marked age-dependent changes. These patterns are suggestive of instability in R-L unsigned asymmetries connected to 5D. Thus, ratios that include 5D may correlate with a tendency for high developmental instability and disease susceptibility. Digit 5 ratios have not as yet been the focus of interest that 2D:4D has attracted. Asymmetries of 2D:4D and 3D:5D are not significantly related. Therefore, summing |R-L| for 2D:4D and 3D:5D might combine information from two different patterns of instability. Thus, increasing effect sizes for correlations between unsigned asymmetry and disease severity. With regard to Study II, in our enlarged sample we have confirmed |R-L| digit ratio asymmetries in non-vaccinated individuals to be clinical markers of risk of hospitalization due to COVID-19. These asymmetries could be used in clinical practice as a Clinical Composite Asymmetry - CCA (asymmetries of 2D:4D and 3D:5D) as a tool for identifying subjects at risk of hospitalization due to COVID-19. Regression analysis showed that the index >0.079 may be a prognostic factor for hospitalization of patients with COVID-19, with sensitivity and specificity of 70%. However, to verify the prognostic value of the suggested index further studies based on larger samples in different ethnic groups should be performed.

D-dimers and fibrinogen levels can predict severe and fatal cases of COVID-19. This observation has been replicated in a number of studies (22–28). Patients from our sample, all hospitalized due to COVID-19, also had initial elevated d-dimers and fibrinogen. However, neither cFA or CCA correlated with these variables. The role of platelets in COVID-19 is more complex and less understood. Some authors reported that decreased number of platelets were more commonly associated with severe COVID-19. However, whether thrombocytopenia may influence disease severity or the severity of the disease may decrease platelets, has not been determined (23). Additionally, the non-survivors with COVID-19 had significantly lower platelet count than survivors (24). Interestingly, not all studies replicated platelet counts to be a predictor of COVID-19 mortality (25). In our sample of hospitalized patients with severe disease the mean platelet count was within normal ranges (normal in 63/91 patients). However, this variable correlated with CCA in non-parametric analysis and with asymmetries of ratios' involving 5D: i.e., 2D:5D and 4D:5D in parametric analysis independent of age, sex and BMI. This may indicate that the index, which was verified as a predictor of hospitalization risk, may not be indicative of mortality risk as it did not correlate with mortality predictors (d-dimers, fibrinogen, white blood count, procalcitonin). However, its correlation with platelet count may be interpreted in the light of a complex role of platelets in COVID-19. Some analyses support the idea that platelets are normal or even elevated in patients with thrombosis/microthrombosis (26). In this regard, higher digit ratio asymmetries in patients hospitalized due to COVID-19 could be indicative of higher values of platelets and tendency to thrombosis.

There are only a few studies in humans examining the role of asymmetry as a marker of an individual's biological condition with its link to immune system functioning (29, 30). Such correlation may result from the fact that poor biological condition is associated with both high asymmetry and high susceptibility to pathogens due to immune system malfunction. To our knowledge, our study is one of just a few covering this issue and the second involving COVID-19 patients focusing on hand asymmetry and their correlation with risk of hospitalization and clinical parameters. Our findings have shown that differences between patients and controls for asymmetry in digit ratios were greatest for 2D:4D, 2D:5D, 3D:5D and 4D:5D. This indicates that perturbations in the growth and asymmetry of (mainly) 5D are markers for elevated probability of hospitalization from COVID-19. In this regard, Manning (19) has reported that digit ratios that included 5D show the highest rate of instability during rapid growth across ages 2 years to 18 years. It was suggested that 5D ratios may be particularly susceptible to perturbations by environmental stressors during childhood and puberty (19). The sum of 2D:4D and 3D:5D asymmetries, which constituted CCA over 0.079 was a good predictor of hospitalization due to COVID-19 in our non-vaccinated population. The results did not support the role of digit ratio asymmetries as indicators of disease severity. However, the observed positive correlation with platelet count needs further studies focused on other indicators of the risk of thrombosis.

The main limitation of the study is that the data were collected before the vaccination program. Therefore, it would be of interest to determine whether vaccinated patients who are hospitalized have particularly high digit ratio asymmetries. Moreover, the results may be useful in case of future pandemics. High rates of hospitalization can cause the collapse of health services. Therefore, it is important to identify subjects especially vulnerable to infections and those who might require hospital care.

In conclusion, |R-L| asymmetries of digit ratios that included 5D showed means and SD's and age-dependent instability that were higher than asymmetries that did not include 5D. Digit ratio asymmetries that included 5D and 2D:4D were elevated in patients hospitalized due to COVID-19. This suggested that markers of developmental instability in the digits are linked to hospitalization for COVID-19. A “Clinical Composite Asymmetry” (composite asymmetry of 2D:4D plus 3D:5D) was verified as a potentially useful marker in identifying individuals who have experienced high developmental instability and are likely to need hospitalization for COVID-19. The index higher than 0.079 discriminated hospitalized patients with a sensitivity and specificity of 70% increasing the risk of hospitalization because of COVID-19 2.5 times. Additionally, a “Clinical Composite Asymmetry” correlated positively with platelet count, but not with other laboratory parameters as well as severity of the disease. Its positive correlation with platelet count needs to be further investigated in patients with elevated platelet counts. Our findings may be useful in identifying subjects endangered with severe course of COVID-19 infection (requiring hospitalization) and they may enable targeted public health measures to avoid their infection and its consequences (e.g., by information campaigns concerning vaccinations against COVID-19).

## Data availability statement

The raw data supporting the conclusions of this article will be made available by the authors, without undue reservation.

## Ethics statement

The studies involving human participants were reviewed and the protocol was agreed by the BioEthical Committee of the Medical University of Lodz (RNN/152/20/KE). Written informed consent to participate in this study was provided by the participants' legal guardian/next of kin.

## Author contributions

JM: conception, drafting the work, analysis of data, and acquisition of the data. AK-T: conception, drafting the work, acquisition and interpretation of data for the work, and review and editing. MJ, JB-W, and OK: acquisition and interpretation of data for the work. BA and AH: conception, supervision, and project administration. All authors contributed to the article and approved the submitted version.

## Conflict of interest

The authors declare that the research was conducted in the absence of any commercial or financial relationships that could be construed as a potential conflict of interest.

## Publisher's note

All claims expressed in this article are solely those of the authors and do not necessarily represent those of their affiliated organizations, or those of the publisher, the editors and the reviewers. Any product that may be evaluated in this article, or claim that may be made by its manufacturer, is not guaranteed or endorsed by the publisher.

## References

[1] Kasielska-Trojan A Manning JT Jabłkowski M Białkowska-Warzecha J Hirschberg AL Antoszewski B. Digit ratios and their asymmetries as risk factors of developmental instability and hospitalization for COVID-19. Sci Rep. (2022) 12:4573. 10.1038/s41598-022-08646-7 35301404 PMC8931101

[2] Manning JT Scutt D Lewis-Jones DI. Developmental stability, ejaculate size, and sperm quality in men. Evol Hum Behav. (1998) 19:273–82. 10.1016/S1090-5138(98)00024-5

[3] Moller A Soler M Thornhill R. Breast asymmetry, sexual selection, and human reproductive success. Ethol Sociobiol. (1995) 16:207–19. 10.1016/0162-3095(95)00002-3

[4] Manning JT Kourkourakis K Brodie DA. Fluctuating asymmetry, metabolic rate and sexual selection in human males. Evol Hum Behav. (1997) 18:15–21. 10.1016/S1090-5138(96)00072-4

[5] Longman DP Oyama S Cracknell J Thompson N Gordon D Stock JT. Fluctuating asymmetry, a marker of poor growth quality, is associated with adult male metabolic rate. Am J Phys Anthropol. (2021) 175:646–55. 10.1002/ajpa.24276 33768527

[6] Manning JT. Fluctuating asymmetry and body weight in men and women: implications for sexual selection. Ethol Sociobiol. (1995) 16:145–53. 10.1016/0162-3095(94)00074-H

[7] Milne BJ Belsky J Poulton R Thomson WM Caspid A Kieser J. Fluctuating asymmetry and physical health among young adults. Evol Hum Behav. (2003) 24:53–63. 10.1016/S1090-5138(02)00120-4

[8] Mellor C. Dermatoglyphic evidence of fluctuating asymmetry in schizophrenia. Brit J Psychiat. (1992) 160:467–72. 10.1192/bjp.160.4.467 1571744

[9] Burton C Stevenson JC Williams DC Everson PM Mahoney ER Trimble JE. Attention-deficit hyperactivity disorder (AD/HD) and fluctuating asymmetry in a college sample: An exploratory study. Am J Hum Biol. (2003) 15:601–19. 10.1002/ajhb.10179 12953172

[10] Naugler CT Ludman MD. Fluctuating asymmetry and disorders of developmental origins. Am J Med Genet. (1996) 66:15–20. 10.1002/(SICI)1096-8628(19961202)66:1<15::AID-AJMG4>3.0.CO;2-V 8957504

[11] Barden HS. Fluctuating dental asymmetry: a measure of developmental instability in Down syndrome. Am J Phys Anthropol. (1980) 52:169–73. 10.1002/ajpa.1330520203 6445164

[12] Manning JT Scutt D Whitehouse GH Leinster SJ. Breast asymmetry and phenotypic quality in women. Evol Hum Behav. (1997) 18:223–36. 10.1016/S0162-3095(97)00002-0

[13] Scutt D Manning JT Whitehouse GH Leinster SJ Massey CP. The relationship between breast asymmetry, breast size and the occurrence of breast cancer. Br J Radiol. (1997) 70:1017–21. 10.1259/bjr.70.838.9404205 9404205

[14] Scutt D Lancaster GA Manning JT. Breast asymmetry and predisposition to breast cancer. Breast Cancer Res. (2006) 8:R14. 10.1186/bcr1388 16563179 PMC1557716

[15] Hudson SM Wilkinson LS De Stavola BL dos-Santos-Silva I. Left–right breast asymmetry and risk of screen-detected and interval cancers in a large population-based screening population. Br J Radiol. (2020) 93:20200154. 10.1259/bjr.20200154 32525693 PMC7446006

[16] Thornhill R Møller AP. Developmental stability, disease and medicine. Biol Rev Camb Philos Soc. (1997) 72:497–548. 10.1017/S0006323197005082 9375532

[17] Benderlioglu Z. Fluctuating asymmetry and steroid hormones: a review. Symmetry. (2010) 2:541–53. 10.3390/sym2020541

[18] Wilson JM Manning JT. Fluctuating asymmetry and age in children: evolutionary implications for the control of developmental stability. J Hum Evol. (1996) 30:529–37. 10.1006/jhev.1996.0041

[19] Manning JT. Sex differences and age changes in digit ratios: implications for the use of digit ratios in medicine and biology. In: Handbook of Anthropometry. Springer. (2012). p. 841–51.

[20] Manning JT Callow M Bundred PE. Finger and toe ratios in humans and mice: Implications for the aetiology of diseases influenced by HOX genes. Med Hypotheses. (2003) 60:340–3. 10.1016/S0306-9877(02)00400-0 12581609

[21] Gangestad S Thornhill R. Individual differences in developmental precision and fluctuating asymmetry: a model and its implications. J Evol Biol. (1999) 12:402–16. 10.1046/j.1420-9101.1999.00039.x

[22] Zhan H Chen H Liu C Cheng L Yan S Li H. Diagnostic value of D-dimer in COVID-19: a meta-analysis and meta-regression. Clin Appl Thromb Hemost. (2021) 27:10760296211010976. 10.1177/10760296211010976 33926262 PMC8114749

[23] Bashash D Hosseini-Baharanchi FS Rezaie-Tavirani M Safa M Akbari Dilmaghani N Faranoush M. The Prognostic Value of Thrombocytopenia in COVID-19 patients; a systematic review and meta-analysis. Arch Acad Emerg Med. (2020) 8:e75. 33134971 PMC7587988

[24] Li Q Cao Y Chen L Wu D Yu J Wang H. Hematological features of persons with COVID-19. Leukemia. (2020) 34:2163–72. 10.1038/s41375-020-0910-1 32528042 PMC7289481

[25] Jiang SQ Huang QF Xie WM Lv C Quan XQ. The association between severe COVID-19 and low platelet count: evidence from 31 observational studies involving 7613 participants. Br J Haematol. (2020) 190:e29–33. 10.1111/bjh.16817 32420607

[26] Thachil J. What do monitoring platelet counts in COVID-19 teach us? J Thromb Haemost. (2020) 18:2071–2. 10.1111/jth.14879 32344467 PMC7267313

[27] Barale C Melchionda E Morotti A Russo I. Prothrombotic phenotype in COVID-19: focus on platelets. Int J Mol Sci. (2021) 22:13638. 10.3390/ijms222413638 34948438 PMC8705811

[28] Barrett TJ Bilaloglu S Cornwell M Burgess HM Virginio VW Drenkova K. Platelets contribute to disease severity in COVID-19. J Thromb Haemost. (2021) 19:3139–53. 10.1111/jth.15534 34538015 PMC8646651

[29] Thornhill R Gangestad SW. Facial sexual dimorphism, developmental stability, and susceptibility to disease in men and women. Evol Hum Behav. (2006) 27:131–44. 10.1016/j.evolhumbehav.2005.06.001

[30] Pawlowski B Borkowska B Nowak J Augustyniak D Drulis-Kawa Z. Human body symmetry and immune efficacy in healthy adults. Am J Phys Anthropol. (2018) 167:207–16. 10.1002/ajpa.23617 30238443

